# Complete Gangrene of the Left Kidney

**Published:** 1875-10

**Authors:** 


Complete Gangrene of the Left Kidney.—M. Avezou reports the case of a man aged 79 years, who, while under treatment for a left-side pneumonia, complained of having had for several years pain in the left lumbar region. He had never suffered with nephritic colic, hæmaturia, or difficult micturition; the urine was normal, containing neither albumen nor sugar ; and there had never been any œdema of the legs and feet. In eight days the patient died.

The autopsy showed the existence of hepatization of the left kidney, with some congestion of the right lung, and a healthy condition of the cardiac valves, though there were some atheromatous patches in the aorta.

The right kidney was of firm consistence, and presented to the eye no alteration. The left kidney, however, was black and friable, and had an offensive smell; the surrounding cellular tissue and the walls of the decending colon at the level of the kidney were also dark-colored, but not at all softened. The ureter of this kidney had a small though permeable calibre, while the renal artery was not obliterated in its terminal portion nor any of its branches.

On microscopical examination, both kidneys showed plainly the alterations due to intersitial nephritis, with atrophy of a large number of the glomèrules, there existing no essential difference between the two organs except in the degree of the pathological change. In some of the tubes colliod casts were found, and in certain parts of the left kidney a very small quantity of granular pigmentary bodies was seen. While the specimens were fresh there was discovered a large number of vibriones and small fatty drobs on both kidneys. The microscopist had some doubt as to the real gangrenous nature of these lesions. The spleen was also dark-colored, but the bladder perfectly healthy. *Medical Times.*

